# Piwi-interacting RNAs in cancer: emerging functions and clinical utility

**DOI:** 10.1186/s12943-016-0491-9

**Published:** 2016-01-15

**Authors:** Kevin W. Ng, Christine Anderson, Erin A. Marshall, Brenda C. Minatel, Katey S. S. Enfield, Heather L. Saprunoff, Wan L. Lam, Victor D. Martinez

**Affiliations:** Department of Integrative Oncology, BC Cancer Agency, Vancouver, Canada

**Keywords:** PIWI-interacting RNA, piRNA, PIWI, Cancer, Tissue specificity, Transcriptome, Small RNA, Non-coding RNA, Epigenetics

## Abstract

PIWI-interacting RNAs (piRNAs) are emerging players in cancer genomics. Originally described in the germline, there are over 20,000 piRNA genes in the human genome. In contrast to microRNAs, piRNAs interact with PIWI proteins, another member of the Argonaute family, and function primarily in the nucleus. There, they are involved in the epigenetic silencing of transposable elements in addition to the transcriptional regulation of genes. It has recently been demonstrated that piRNAs are also expressed across a variety of human somatic tissue types in a tissue-specific manner. An increasing number of studies have shown that aberrant piRNA expression is a signature feature across multiple tumour types; however, their specific tumorigenic functions remain unclear. In this article, we discuss the emerging functional roles of piRNAs in a variety of cancers, and highlight their potential clinical utilities.

## Background

**Fig. 1:**
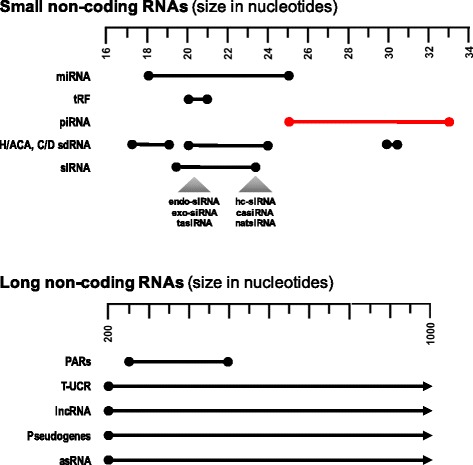
Selected types of small and long non-coding RNAs. Non-coding RNAs show diversity in size and function. The small non-coding RNA category (20–35 bp) includes: microRNAs (miRNAs), tRNA-derived fragment (tRF), PIWI-interacting RNAs (piRNAs), C/D snoRNA-derived RNA (sdRNAs), and small interfering RNAs (siRNAs). The long non-coding RNA category (>200 bp) includes promoter associated long RNAs (PARs), transcribed ultraconserved regions (T-UCR), long non-coding RNAs (lncRNAs), pseudogenes, and antisense RNAs (asRNAs). piRNAs (red line) are larger than other small RNAs and, unlike most of the other small RNA (with the exception of snoRNA), are functionally active both the nucleous and the cytoplasm

With the realization that less than 3 % of the transcribed human genome is translated into protein, there has been a surge of interest in the role of the non-coding RNA transcriptome and its contribution to pathogenesis [1–4]. Among the types of human non-coding RNAs, microRNAs (miRNAs) and long non-coding RNAs (lncRNAs) have been extensively studied in cancer [5–21] (Fig. 1). P-element-induced wimpy testis (PIWI)-interacting RNAs (piRNAs) are a class of small non-coding RNA molecules that have been recently recognized to be relevant to cancer biology. Today, conservative estimates for the total number of piRNAs in the eukaryotic genome parallel those of protein-coding genes (~20,000), and largely exceeds the estimated 2,000 miRNA loci present [22, 23]. Initially termed “repeat associated small interfering RNAs” (rasiRNAs) [24], they were designated piRNAs for their interaction with the PIWI subfamily of Argonaute proteins [25, 26]. PIWI proteins have been extensively explored in germline and stem cells, and have the unique ability of binding 2′-*O*-methylated RNAs -a distinguishing feature of piRNAs- at their 3′ end [27–29].

The piRNA/PIWI interaction is an example of the highly conserved small RNA-guided mechanism of gene regulation, which involves interaction between a sequence-recognition RNA and a member of the Argonaute protein family [30]. The specific mechanism by which regulation occurs is dependent on the type of small RNA. Small interfering RNAs (siRNAs) and miRNA interact with the Ago subfamily of Argonaute family, whereas piRNAs interact with PIWI proteins [31, 32]. Furthermore, unlike siRNA and miRNA, the piRNA/PIWI complex has been primarily described as functioning through epigenetic silencing rather than transcript targeting [33].

PIWI was first discovered in *Drosophila melanogaster* in relation to germline stem cell self-renewal; therefore, much of the focus has been on the piRNA/PIWI complex’s role in the germline [34]. Germline piRNAs are primarily involved in the silencing of selfish genetic elements, including transposable elements (TEs) [26]. In addition to their well-known epigenetic functions [33, 35], recent studies in *Drosophila* and mice suggest that piRNAs may additionally regulate gene expression through a miRNA-like transcript silencing mechanism in the cytoplasm [36–38].

In this review, we discuss what is known about piRNAs in germline cells, and their emerging roles in somatic and cancerous tissues. Furthermore, we discuss the diagnostic and prognostic potential utility of piRNAs.

## The piRNA/PIWI protein interaction in germline and somatic tissues

**Fig. 2:**
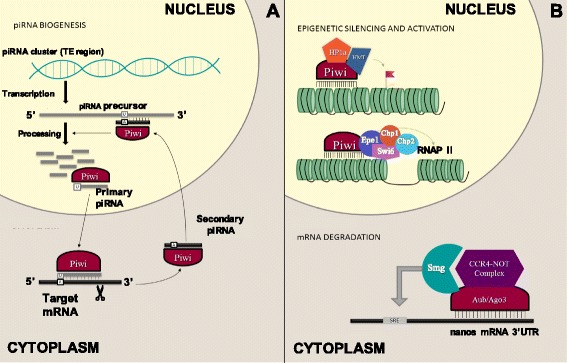
Biogenesis and functions of the piRNA/PIWI complex. piRNA and PIWI proteins form a ribonucleoprotein complex that is primarily responsible for the maintenance of genome integrity through transposable element (TE) silencing in the germline at both the transcriptional and post-transcriptional level. **a** The ribonucleoprotein complex is active in piRNA biogenesis, where it cleaves target RNAs at the position 10 and 11 of the guide strand, generating the 5′ end of the cleavage product that will be loaded to a second PIWI protein, and gives rise to a secondary piRNA, after nucleolytic processing of the 3′ end. Primary piRNAs have uridine (U) bias at their 5′ nucleic acid, while secondary piRNAs, which shows 10 nucleotide complementarity with primary piRNAs at their 5′ ends, exhibits a bias for adenosine (A) at the tenth nucleotide. **b** In the nucleus, the complex is active in epigenetic silencing, through the establishment of a repressive chromatin state as a result of the recruitment of Heterochromatin protein 1 (HP1a) and histone methyltransferases (HMT); and epigenetic activation, through euchromatic histone modifications that allows binding of proteins such as JmjC domain-containing histone demethylation protein 1 (Epe1), chromodomain protein1 and 2 (Chp1, Chp2) and Chromatin-associated protein Swi6. In the cytoplasm, it is active in mRNA degradation through association with the carbon catabolite repressed 4 - negative on TATA-less (CCR4–NOT) deadenylation complex, together with Smaug (Smg)

piRNAs accomplish their regulatory function through binding to PIWI proteins from the Argonaute family, resulting in the formation of a silencing ribonucleoprotein complex that can recognize and silence complementary sequences [39, 40]. The most well-established function of this complex is the maintenance of genome integrity through TE silencing in the germline at both the transcriptional and post-transcriptional level, which is linked to its biogenesis and is a conserved function among different animal species [41, 42] (Fig. 2). However, it has also been suggested that piRNAs have specific functions in both the germline and somatic tissues of various organisms, including conserved mammalian biological functions [43].

### piRNA function in germline tissue

#### Repression of transposable elements

TEs can contribute to genetic diversity as well as genetic instability [44, 45]. This can result in pathogenesis through gene deregulation, chromosome rearrangement, and deleterious mutation, and has been linked to a number of cancers [46, 47]. In particular, the role of non-long terminal repeat (non-LTR) TEs, including long interspersed elements (LINEs) and short interspersed elements (SINEs) has been implicated, with particular emphasis on non-LTR families L1, SVA, and Alu in leukemia, breast, ovarian, and colon cancers [48]. In particular, somatic L1 insertions have been found to occur in and disrupt the expression of commonly mutated genes in cancer [47]. This enhanced L1 insertion has also been demonstrated in vitro in a variety of cancer cell lines, which additionally display increased RNA polymerase II binding to TEs compared to non-malignant cell lines [49].

The PIWI protein localizes to the nucleus and works at the transcriptional level, establishing a repressive chromatin state as the result of an increase in H3K9me3 and HP1 chromatin marks (Fig. 2b) [41]. Overexpression of piRNA has been associated with the recruitment of PIWI, HP1a, and Su(var)3–9, the reduction of RNAPII, and the enrichment of H3K9me2/3 [33]. Indeed, piRNA-mediated epigenetic silencing of TEs in *Drosophila* has been shown to result in the silencing of adjacent genes as well [50]. In *Drosophila*, it has been shown that the PIWI proteins Aubergine (Aub) and Argonaute 3 (Ago3) localize to the cytoplasm and repress transposons at a post-transcriptional level through the cleavage of transcripts (Fig. 2b) [36, 51]. The coexistence of both cytoplasmic and nuclear piRNAs further supports this theory [37]. Additionally, it has been reported that Ago3-mediated RNA cleavage drives the production of antisense piRNAs that direct transcriptional silencing by Piwi in Drosophila [52].

piRNA/PIWI-mediated regulation of TEs has also been well characterized in mammals. Mice exhibit the expression of three PIWI-like proteins: Miwi, Mili, and Miwi2 [53]. Miwi2 possesses many similarities to the PIWI protein present in *Drosophila* [54] and it has been suggested that both Mili and Miwi2 affect the methylation status of repetitive elements in order to maintain the stable repression of transposons [54, 55]. In mice, specific piRNA knockdown leads to the derepression of LINE-1 [53]. As well, a decrease in piRNA cluster expression has been shown to be associated with increased TE activity [53]. A study of pig testes suggests that a conserved and dynamic mammalian piRNA/PIWI system plays a critical role in post-transcriptional gene regulation [43]. A similar finding occurred in marmoset testes tissue, where small RNA profiling revealed that piRNA clusters may use pseudogene-derived piRNAs to regulate protein-coding genes based on the strand-bias of TE-derived piRNAs [56]. This finding further supports a conserved mammalian piRNA system.

#### Epigenetic activation

In addition to a role in TE activity, piRNAs have also been shown to play a role in the epigenetic activation of gene expression. PIWI protein complexes have been shown to promote euchromatic histone modifications and the transcription of piRNAs [35]. Specifically, PIWI has been shown to have a positive regulatory role in the expression of the *3R-TAS1* piRNA in germline stem cells of *Drosophila melanogaster*. The absence of PIWI has been shown to exert opposite genomic regulatory effects on different parts of the gene, namely resulting in enhanced telomere position effects in the white eye gene in *Drosophila*. Thus, since PIWI is required for the expression of this reporter gene in a dose-dependent manner, it is shown to promote the active epigenetic state of *3R-TAS* [35]. Additionally, in *Caenorhabditis elegans*, the piRNA and Argonaute protein CSR-1 complex has been shown to cause additional recruitment of histone modifying enzymes, which result in epigenetic activation [57].

#### mRNA decay

In murine spermatogenesis, piRNAs play key silencing roles beyond TE repression. Following the first wave of TE-derived piRNA production, a second wave of piRNA has been observed to target transcripts in late stages of spermatogenesis [58]. Through the recruitment of Miwi and deadenylase CAF1, a piRNA-induced silencing complex is assembled and induces mRNA deadenylation and degradation [58]. This miRNA-like mechanism has been shown to be dependent on the physical association of Miwi with piRNA target sites on mRNA [59].

### piRNA expression and functions in somatic tissue

Although most attention has been given to the functional role of piRNAs in germline TE regulation, increasing evidence has pointed to piRNA function not only beyond TE regulation, but also outside germline cells. Studies in *Drosophila* and mammals suggest a role in gene regulation performed by piRNAs derived from protein-coding genes [43]. Colocalization of piRNAs encoded by mitochondrial DNA and PIWI proteins in mammalian somatic cell lines suggests they may function as part of the stress response [60]. Additionally, the Smg-CCR4-NOT-Aub-dependent mechanism of mRNA deadenylation and decay has also been demonstrated to occur in the soma in *Drosophila* [51, 61].

Consistent with emerging roles for piRNAs in the cytoplasm as well as in the nucleus, piRNA-like-163 (piR-L-163) was observed to localize to both the cytoplasm and nucleus of human bronchial epithelial cells (HBECs) at various points throughout the cell cycle [62]. Analysis of piR-L-163-interacting proteins through liquid chromatography-mass spectrometry (LC-MS) implicated the ERM (Ezrin/Radixin/Moesin) family of proteins. Strikingly, piR-L-163 appeared to bind exclusively to activated phosphorylated ERM proteins and not to inactive forms, and was also demonstrated to be critical for ERM’s biological function [62].

Though exploration of piRNA expression in humans has focused mainly on germline tissue, it has recently been noted that piRNA are expressed across human somatic tissue. In human adult testes, expressed piRNAs are enriched in 3′ untranslated regions UTRs, as compared to coding DNA sequences (CDSs) and 5′UTRs [63]. Additionally, it has been shown that the number of piRNAs expressed in somatic tissue is considerably lower than that observed in germline tissues, although there is tissue specificity associated with the expressed piRNAs [64].

## piRNA expression in humans: novel functions and role in cancer

### Functional roles of piRNAs in cancer

**Fig. 3:**
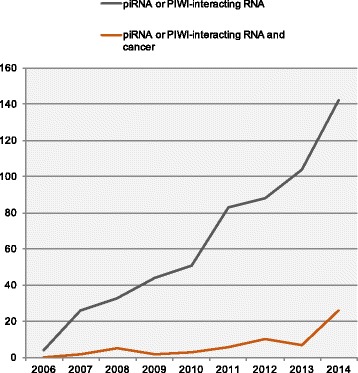
Number of piRNA-related publications per year. Search was performed within EndNote (version 7, Thomson Reuters) and manually filtered. Number of entries are based on a United States National Library of Medicine (NLM) PubMed search using the terms “piRNA OR PIWI-interacting RNA” (blue line) or “piRNA OR PIWI-interacting RNA AND cancer” (red line) and annual (Jan 1-Dec 31) date limitations. 2015 publications were not included in the search

As piRNAs are implicated in gene regulation and silencing, there has been a budding interest in defining the role of piRNAs in human disease [65]. In particular, interest in the piRNA field has focused on human cancers, as demonstrated in a recent surge of publications (Fig. 3). In conjunction with this, the role of PIWI proteins themselves in cancer has been extensively studied [66], further underscoring the role of the piRNA/PIWI complex in tumorigenesis.

Additionally, over or underexpression of piRNAs that target mRNA transcripts (e.g. those containing transposon-derived sequences in their 3′UTRs) could also play a driver role through degradation or inhibition of tumour suppressor genes or oncogenes, respectively [37]. On the other hand, the disruption of piRNAs that normally suppress TE activity may facilitate mutagenic retrotranspositions and genomic instability, thereby contributing to tumorigenesis; however, this does not appear to be the case as ectopic expression of piRNA/PIWI tends to result in a more aggressive cancer phenotype [67].

Epigenetic modifications, such as DNA hypo/hypermethylation and histone modifications, are hallmarks of the development and progression of cancer [68]. Remarkably, the recent functional evidence concerning the role of piRNAs in tumorigenesis points to the involvement of these small RNAs in the regulation of epigenetic mechanisms, coupled with the acquisition of cancer-like phenotypes in human models. It has been proposed that the piRNA/PIWI may contribute to tumorigenesis through aberrant DNA methylation resulting in genomic silencing and promotion of a “stem-like” state [69]. More specifically, piRNAs are able to induce DNA methylation changes in genomic regions close to their binding sites. In human lymphoma and breast cancer cell lines, single-copy piRNAs mediate gene-specific DNA methylation through imperfect binding to genomic DNA near target CpGs at non-TE sites [70]. Additionally, breast cancer cell lines transfected with a variant of piR-021285 containing the rs1326306 SNP showed reduced methylation at a CpG site within the 5′ UTR/first exon of ARHGAP11A gene, resulting in increased gene expression and augmented migration capability in cell lines compared to those carrying the WT form of this piRNA [71].

piRNAs can also activate the gene expression by inducing euchromatin state through upregulation of H3K4me3 and inhibition of H3K27me3 in subtelomeric heterochromatin regions in *Drosophila* [35]. Consistent with this mechanism, piRNA-like molecules located in intronic regions of the Growth Arrest Specific 5 (GAS5) gene, together with PIWIL1/4, are able to recruit a hCOMPASS-like complex to the promoter region of the Tumor Necrosis Factor (Ligand) Superfamily, Member 10 (TRAIL) tumor suppressor gene. This, together with induction of H3K4 methylation and H3K27 demethylation, resulted in transcriptional activation of TRAIL and consequently, inhibition of tumor growth [72].

piRNAs have been also linked to other possible tumorigenic, non-epigenetic functions in cells, such as cell cycle regulation. Depletion of the piRNA piR-L-163 resulted in accelerated DNA synthesis and G2-M accumulation, together with increased invasion and cell migration capabilities in human bronchial epithelium (HBE) cell lines [62]. This occurs through specific binding of piR-L-163 to the phosphorylated form of the ERM (ezrin, radixin and moesin) protein, which suggests a functional role of piRNAs in lung tumorigenesis. Remarkably, this also reveals another dimension of PIWI-independent functional roles of piRNAs in cancer.

### Altered expression of piRNAs in cancers

Despite the number of total piRNAs encoded in the human genome, only a small fraction is consistently expressed in both non-malignant and tumor tissue [64]. Aberrant expression of the piRNA/PIWI complex and its correlation with clinical features in malignant tissues points to a role for piRNAs in cancer. However, this role is uncertain as the function of piRNA in somatic tissue is still being established.

**Fig. 4:**
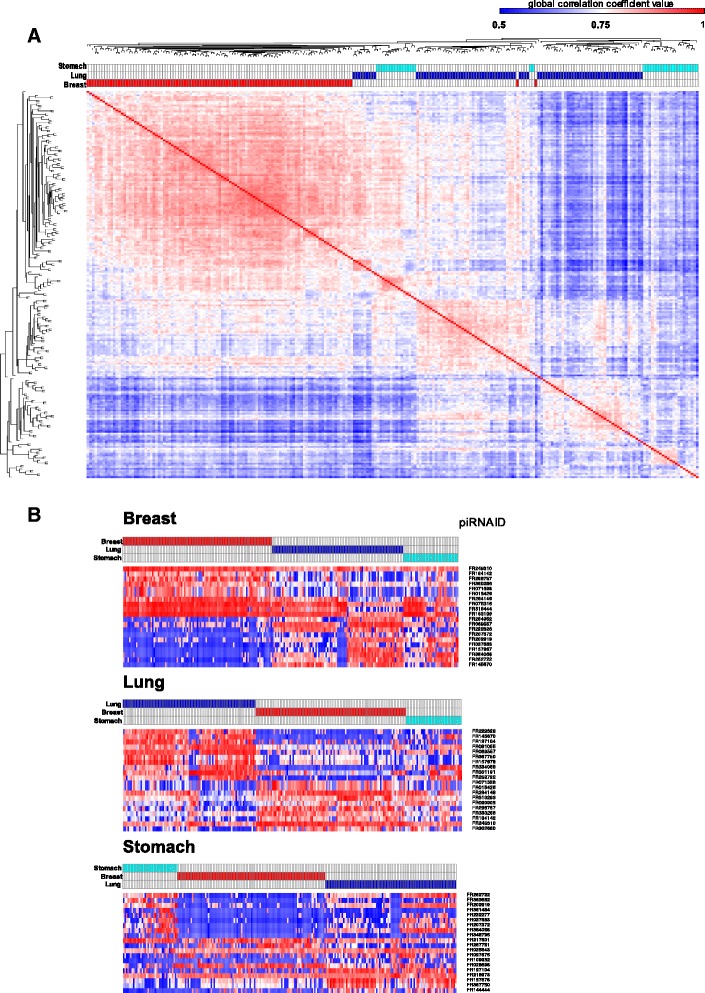
piRNA expression pattern across human somatic, non-malignant tissues. **a** As an example of piRNA expression pattern heterogeneity across human somatic tissues, a similarity matrix was constructed using rank-normalized piRNA expression levels from breast (red), lung (blue) and stomach (turquoise) non-malignant human tissue. This data is derived from small RNA sequencing efforts from the The Cancer Genome Atlas (TCGA) consortium and processed through a computational pipeline to detect piRNA expression. Briefly, sequencing reads with a higher fraction of piRNAs (24–31 bp length) were mapped to the hg19/GRCh37 built. Aligned reads were subsequently quantified using specific piRNAs annotations, which account for the genomic locations of piRNA-encoding loci. Sample clustering was performed using Pearson correlation, and the correlation coefficient is color-coded from blue (low correlation) to red (high correlation). Noticeably, different degrees of overall correlation across samples from the same tissue origin are observed. Samples derived from breast tissue are mostly clustered together, denoting a high degree of similarity of piRNA expression patterns, while lung and stomach denotes subgroups. The molecular origin and physiological functions of these piRNA-defined subgroups is not completely understood, although it has been shown that these subgroups are linked to specific clinical features in cancer patients. **b** A subset of piRNAs is able to define a tissue-specific piRNA expression signature. In the figure, the top 10 over- and under-expressed piRNA on each tissue is compared against others. The expression is significantly different, assessed by comparative marker selection tool, with false discovery-rate (FDR) corrected p-value ≤ 0.05. Top panel: breast vs. lung + stomach; middle panel: lung vs. breast + stomach; bottom panel: stomach vs. breast + lung

A recent study revealed that piRNA expression patterns in human somatic tissue are markedly different across tissue types [64]. Different degrees of heterogeneity in expression patterns can be observed across tissues. For example, when the expression profiles of non-malignant tissue derived from breast, lung and stomach are compared, we observe that breast tissue shows a highly correlated pattern across samples from different individuals. On the other hand, discrete clusters of samples can be observed in lung and stomach tissue (Fig. 4). Additionally, tissue-specific piRNA signatures are also evident. The underlying cause for these differences are still unknown; however, these results warrant further exploration to elucidate whether these differences might be linked to tissue-specific features.

**Fig. 5:**
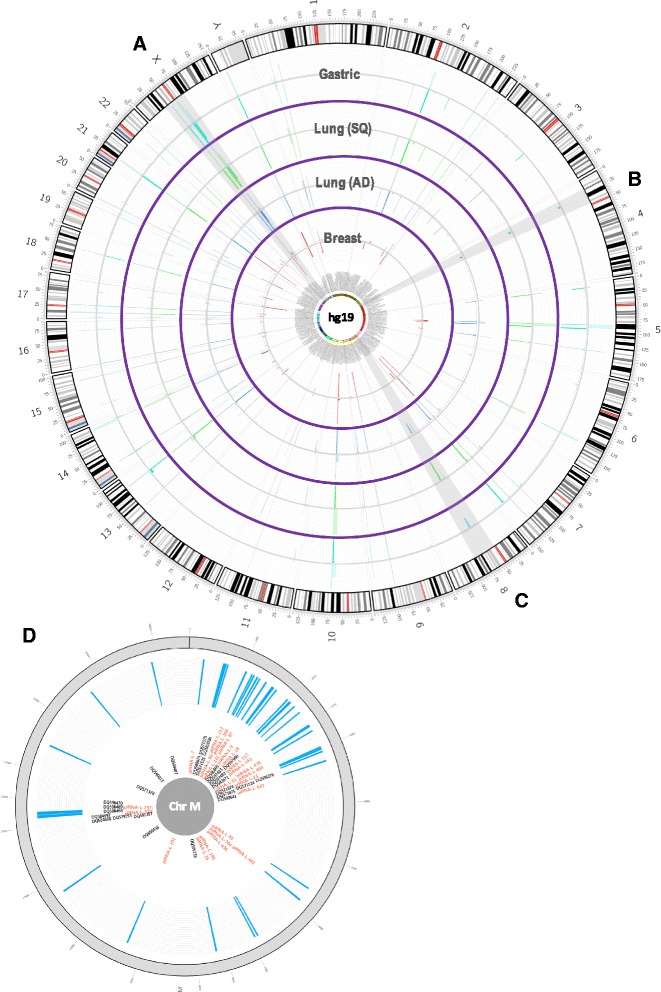
Distribution of piRNAs expression in human cancer genome. Circular representation of the human genome (chromosomes 1–22, X and Y). The distribution of piRNA expression in non-malignant (outward lines) and tumors (inward lines) from breast (red), lung (blue and green from samples derived from adenocarcinomas and squamous cell carcinomas, respectively) and stomach (turquoise) is shown in a circular representation of the human genome. Clusters of highly expressed piRNAs are evident in chromosomes 2, 5, 10, 13, 21 and X. Although expression of some piRNAs is common across tissues (**a**), some of them show tissue-specific features (**b**). Moreover, differences between tumour and normal tissue are observable (**c**). The outer grey circle is a representation of the human mitochondrial genome. Blue bars depict the location of of these piRNA species in the mitochondrial DNA (note: the height of the blue bars does not represent expression levels of the piRNA species, since data for some of the original studies is not still publically available. Inner black and red labels represent previously known piRNA sequences and piRNA-like species that map to the mitochondrial genome [59, 61] (**d**)

The aberrant expression of a small number of piRNAs has been identified in multiple myeloma (MM), breast, lung, gastric and other cancers (Table 1, Fig. 5) [64]. However, findings in liver, pancreas, MM, and lung remain preliminary. As well, few studies have addressed the functional role of piRNAs in cancer progression.

**Table 1:** Summary of altered piRNA expression in cancer

Cancer Type	piRNA ID	Alteration	Detection method	Ref.
Breast	piR-4987	upregulated	deep sequencing of matched tumour-normal tissues, validated via PCR	[73]
	piR-20365			
	piR-20485			
	piR-20582			
	piR-34736	downregulated	small RNAseq of cell lines and tumour biopsies (PCR validation of piRNA biogenesis proteins only)	[74]
	piR-36249			
	piR-35407			
	piR-36318			
	piR-34377			
	piR-36743	upregulated		
	piR-36026			
	piR-31106			
Breast (CD44+/CD24−)	piR-932	upregulated	piRNA microarray of tumour tissue	[75]
Gastric	piR-823	downregulated	piRNA microarray of cell lines, validated via PCR	[76]
	piR-651^a^	upregulated	piRNA microarray of matched tumour-normal tissues, validated via PCR	[77]
	piR-59056	upregulated	Small RNAseq of matched tumour and normal tissues	[78]
	piR-32105			
	piR-58099			
Kidney	piR-32051	upregulated	Small RNAseq of tumour tissue, validated via PCR	[79]
	piR-39894			
	piR-43607			
	piR-57125	downregulated in non-metastatic vs normal; downregulated in metastatic vs non-metastatic	piRNA microarray of matched tumour-normal tissues, validated via PCR	[80]
	piR-38756	downregulated in non-metastatic vs normal; upregulated in metastatic vs non-metastatic		
	piR-30924			
Liver	piR-Hep1	upregulated	deep sequencing of cell lines, validated in matched tumour-normal tissues via PCR	[81]
Pancreas	piR-017061	downregulated	small RNAseq of tumour and normal tissue	[83]
Multiple Myeloma	piR-823	upregulated	piRNA microarray of tumour and normal tissue, validated via PCR	[84]
Lung	piR-L-163	downregulated	small RNAseq of NSCLC cell lines	[62]

^a^also upregulated in colon, lung, and breast cancer tissue and hepatic carcinoma, mesothelioma, cervical, breast, and lung cancer cell lines

#### Breast cancer

Deep sequencing and RT-PCR of breast cancer tumours and matched normal tissue samples found four significantly upregulated piRNAs: piR-4987, piR-20365, piR-20485, piR-20582 [73]. Additionally, small RNA sequencing from breast cancer cell lines and tumour biopsies identified over 100 piRNAs expressed in breast cancer, some of which were influenced by cell cycle progression and the oncosuppressive estrogen receptor β. Of the 100 piRNAs identified, 30 were differentially expressed between malignant and non-malignant mammary epithelial cells. Eight piRNAs in particular were found to be significantly differentially expressed between tumour and matched non-malignant tissue: piR-34736, piR-36249, piR-35407, piR-36318, piR-34377, piR-36743, piR-36026, and piR-31106. It is noteworthy that these mapped to regions involved in key biological processes involved in cancer. The expression of PIWIL1 and PIWIL4 and other key players in the piRNA biogenesis pathway also suggest a driver role for piRNAs in breast cancer [74]. Another study implicated piR-932 in aberrant DNA methylation. PIWIL2 was found to be significantly overexpressed in breast cancer stem cells, inducing epithelial-to-mesenchymal transition (EMT) following TGF-β1 treatment, and PIWIL2 also formed a complex with one of three significantly overexpressed piRNAs, piR-932. The expression level of putative tumour suppressor Latexin decreased as PIWIL2 expression increased due to promoter region CpG island methylation. This suggests the piR-932/PIWIL2 complex results in hypermethylation of *Latexin*, which in turn promotes EMT [75]. Similarly, a recent study found that piR-021285 is involved in methylation at a number of known breast cancer-related genes. In particular, the single nucleotide polymorphism (SNP) rs1326306-containing piR-021285 was found to increase mRNA expression of *ARHGAP11A* and invasiveness in an *in vitro* cell line model [71].

#### Gastric cancer

piR-823 was found to be significantly downregulated compared to matched non-malignant samples in four patients. Cancer cell growth was inhibited by overexpression of piR-823 in vitro, and tumour growth in a xenograft model was significantly suppressed, both in a dose-dependent manner. As there was no association with clinicopathological features of patients, its oncogenic role in gastric cancer has been suggested to be during pre-cancerous stages of tumorigenesis [76]. Aberrant expression of another piRNA, piR-651, was identified in gastric cancer. Significant overexpression of piR-651 was found in 4 paired tumour and non-malignant samples by piRNA microarray and confirmed by PCR in a larger cohort of tumour and non-malignant tissues. piR-651 inhibition in gastric cancer cell lines suppressed growth in a dose dependent manner and resulted in cell cycle arrest at the G_2_/M stage [77]. Upregulation of piR-651 was also observed in colon, lung, and breast cancer tissues as well as hepatic carcinoma, mesothelioma, cervical, breast, and lung cancer cell lines suggesting an oncogenic role across cancer types [77]. More recently, small RNA sequencing data from 320 gastric cancer and 38 non-malignant tissue samples identified the expression of 312 piRNAs, of which half were significantly differentially expressed in tumour tissues compared to non-malignant tissues. Some exhibited either gastric cancer or non-malignant stomach tissue specific expression; most were overexpressed in tumour tissue, including piR-59056, piR-32105, and piR-58099 [78].

#### Kidney cancer

In kidney cancer, deep sequencing of 24 samples found 19 piRNAs to be differentially expressed between clear cell renal cell carcinoma (ccRCC) and benign kidney tissue, and 46 piRNAs to be differentially expressed between localized and metastatic ccRCC. Of those piRNAs aberrantly expressed in metastatic ccRCC, three upregulated piRNAs, piR-32051, piR-39894, piR-43607, are from the same piRNA cluster on chromosome 17, and were also overexpressed in embryonic and neoplastic kidney cell lines compared to benign [79]. Validation in an external cohort also found them to be associated with ccRCC metastasis as well as high tumour stage and patient survival [79]. Another study on ccRCC, aimed at identifying prognostic markers, identified 235 upregulated and 369 downregulated piRNAs in malignant tissue by piRNA microarray from 106 patient samples. RT-qPCR analysis of confirmed decreased expression of piR-38756, piR-57125, and piR-30924 in non-metastatic primary tumours compared to normal tissue. However, when comparing metastatic primary tumours and bone metastases to non-metastatic primary tumours, piR-57125 was downregulated and piR-38756 and piR-30924 were upregulated [80].

#### Liver cancer

A novel piRNA, piR-Hep1, was identified through deep sequencing and found to be upregulated in hepatocellular carcinoma (HCC) compared to adjacent non-malignant liver tissue. PIWIL2 expression, but not PIWIL4 expression, positively correlated with expression of piR-Hep1 in HCC, suggesting PIWIL2 complexes with piR-Hep1 to promote tumorigenesis. piR-Hep1 silencing inhibited cell viability, motility, and invasiveness and also reduced levels of active phosphorylated AKT [81], which has been previously shown to be activated by PIWIL2 inhibiting apoptosis through the Stat3/Bcl-X_L_ pathway [82].

#### Pancreatic cancer

Next generation sequencing revealed one piRNA, piR-017061, located within HBII-296A snoRNA, to be significantly downregulated in pancreatic ductal adenocarcinoma (PDAC) compared to non-malignant pancreatic control tissues [83].

#### Multiple myeloma

In contrast to gastric cancer, in which it was downregulated, piR-823 was found to be upregulated in MM patients and MM cell lines; overexpression correlated with worse prognosis. *In vivo*, piR-823 inhibition resulted in differential expression and phosphorylation of key apoptotic, proliferative, and cell cycle genes and also suppressed pro-angiogenic activity. It was also shown to be a direct regulator of DNA methyltransferases 3A and 3B. The inhibition of piR-823 decreased global methylation in MM cells, suggesting that aberrant DNA methylation targeting tumour suppressor genes promotes tumorigenesis. Decreased tumour size with piR-823 inhibition in xenograft mouse models further supports its role in tumour growth [84].

#### Lung cancer

A recent lung cancer study identified 555 piRNAs expressed in 8 non-small cell lung cancer (NSCLC) and 3 human bronchial epithelial cell (HBEC) lines, of which over half were novel piRNA-like (piRNA-L) sncRNAs [62]. These piRNAs were found to be differentially expressed between NSCLC and HBEC lines, and also among adenocarcinoma and squamous cell carcinoma subtypes. The most commonly downregulated piRNA in NSCLC was piR-L-163, located in intron 10 of the *LAMC2* gene. piR-L-163 was implicated in cell cycle regulation, as inhibition enhanced cell viability and proliferation and accelerated DNA synthesis and G_2_-M accumulation compared to the control. Blocking piR-L-163 also significantly increased invasion and migration of HBECs. This piRNA-L localized to the cytoplasm and was shown to bind to phosphorylated ERM proteins. piR-L-163 directly regulates protein function as this binding interaction was essential for p-moesin to bind F-actin and EBP50 [62].

Aberrant expression of piRNA and piRNA pathway components are observed across cancer types and have been shown to influence hallmarks of cancer. While differences in piRNA expression are capable of distinguishing tissues types -possibly indicating a tissue-specific role for these piRNAs- some piRNAs are consistently highly expressed across all tissue types examined, which could suggest a conserved general function for these piRNAs in somatic tissues [64]. Though functional studies are still preliminary, it is clear that the mechanisms through which piRNA contribute to cancer are varied owing to the diverse functionality of piRNAs in germline, and now somatic, tissues.

## Diagnostic and prognostic utility of piRNAs

As diagnostic and therapeutic procedures move from biopsies towards less invasive methods, piRNAs represent an attractive biomarker candidate. Much of this research has centred around circulating nucleic acids in plasma. As with miRNA, piRNAs remain largely undegraded in circulation and have the ability to resist a wide range of incubation and storage conditions regularly used in the laboratory [85, 86]. Indeed, a recent study of piRNAs in gastric cancer found that, when compared to an existing miRNA-based biomarker detection system, piRNAs provided higher sensitivity and specificity [87]. One caveat with piRNA-based biomarkers is that, as with miRNA, normalization across subjects has proved challenging; however, a recent study has described the potential of salivary piRNA being used as an alternative to or in conjunction with plasma-based systems [88]. In particular, the high abundance of piRNA in saliva as well as high concordancy levels between subjects warrants further exploration. Differential or cancer-specific piRNA expression has been reported across a number of tumour types (Table 2). In gastric cancer, a three-piRNA signature of piR-59056, piR-54878, and piR-62701 was able to effectively separate patients by risk of recurrence. A similar association with recurrence-free survival was observed in colon cancer [78]. Both piR-823 and piR-651 have been investigated as potential plasma biomarkers for gastric cancer. Compared to healthy controls, both were found at significantly lower levels in peripheral blood in cancer patients. Additionally, piR-823 and piR-651 levels in peripheral blood were found to associate with tumour stage and distant metastasis, and were also able to effectively distinguish gastric adenocarcinoma from signet ring cell carcinoma [87]. In another study, tumour-derived levels of piR-651 were found to associate with TNM stage and poorly differentiated tumours [77].

**Table 2:** piRNAs associated with clinical parameters in cancer

Cancer Type	piRNA ID	Clinical Parameter	Reference
Gastric	piR-59056	Recurrence-free survival (3 piRNA signature)	[78]
	piR-54878		
	piR-62701		
	piR-651	TNM stage	[77]
	piR-651	T stage, metastasis, gastric vs. signet ring cell (2 piRNA signature)	[87]
	piR-823		
Colon	piR-59056	Recurrence-free survival (3 piRNA signature)	[78]
	piR-54878		
	piR-62701		
Breast	piR-4987	Lymph node positivity	[73]
Kidney	piR-30924	Overall survival, tumour progression (3 piRNA signature)	[80]
	piR-57125		
	piR-38756		
	piR-32051	Cancer-specific survival, stage, metastasis (3 piRNA signature)	[79]
	piR-39894		
	piR-43607		

Though the role of piRNAs in breast cancer is less well-described compared to that in gastric cancer, recent studies have demonstrated that they too can be effectively harnessed for clinical purposes. As with in gastric cancer, a high proportion of all piRNAs expressed in breast cancer map to protein coding regions in the genome, primarily within intragenic regions [74]. A study of 54 breast ductal carcinoma tissue samples identified six piRNAs that were differentially expressed - among them, piR-4987 upregulation was associated with lymph node metastasis [73]. Similarly, two recent studies done in clear cell renal carcinoma implicated piRNAs as being of important prognostic value: Busch et al. identified three piRNAs significantly associated with overall survival and tumour progression, two of which were able to serve as independent prognostic markers [80], and Li et al. described a three-piRNA signature located on chromosome 17 that was shown to be associated with metastasis, stage, and cancer-specific survival [79]. These advances highlight the potential of piRNA-based prognostic markers across a variety of tumour types.

Beyond a biomarker role, a recent study demonstrated the potential for piRNA as a therapeutic tool. In a mouse model, it was found that artificial piRNA could be generated through the expression of sense and antisense transcripts, which was sufficient to induce epigenetic silencing of the target gene [89]. This research highlights the potential of piRNAs as clinical biomarkers as well as reinforces the need to further understand piRNA mechanisms of action for potential therapeutic exploitation.

## Conclusions and future directions

The advent of high-throughput sequencing technologies has effectively enabled the delineation of the crucial roles of non-coding RNA in pathogenesis as well as in normal cellular biology. In particular, piRNAs have emerged as a highly promising area of study given their gene regulatory function both in the nucleus and in the cytoplasm. Further study into the distinct splicing mechanisms involved in piRNA production is also warranted to determine whether there is a link between method of production and function.

While expression patterns of other small non-coding RNAs such as miRNAs have been extensively studied, malignant and non-malignant piRNA expression patterns are largely uncharted. Aberrant piRNA expression has now been extensively described across a variety of tumour types, though further research is needed to elucidate the specific oncogenic or tumour-suppressing mechanisms. Moreover, the expression patterns are specific to malignancies, and subgroup-specific piRNA expression patterns from tumours of the same organ exist and correlate with key clinical features relevant to each tumour type.

The study of distinct alterations affecting piRNA expression and their functional consequences in the development of human cancer is advancing rapidly. While we can still apply lessons learned from the miRNA field, the evidence points towards an additional spectrum of functions potentially affected by piRNAs. More importantly, these functions can be complementary to those performed by other small RNA (e.g. DNA-level coupled with RNA-level regulation of gene expression, performed by piRNAs and miRNAs, respectively). If this phenomenon exists, are functionally-related genes subject to this type of “dual” regulation?. It is also very relevant to investigate the physiological roles of piRNAs in normal somatic tissue. By understanding physiological function, it would be possible to decipher molecular functions potentially affected when piRNA expression is deregulated during cancer development. This knowledge will be also useful to refine therapeutic applications of piRNA.

The interaction between piRNA and PIWI proteins is well established and characterized in a vast amount of organisms. While these interactions may impact tumorigenic functions (supported by the fact that PIWI proteins are deregulated in a variety of cancers), it is also intriguing to decipher other PIWI-independent interactions of piRNAs. The discovery of such novel interactions may lead to the discovery of new mechanisms of regulation of malignancy-associated pathways affected by altered piRNA expression.

The development of piRNAs as therapeutic tools also raises question and challenges. Since recent evidence points to piRNAs as potential tools to modulate the activity of tumor suppressor genes (e.g. TRAIL in breast cancer), it is important to investigate what other tumor suppressor activities can be enhanced by piRNA expression (either natural or ectopic). This is particularly relevant due to the tissue specificity of both piRNA expression and tumor suppressor activity. Further study of the mechanistic role of piRNA in tumour progression across a wider spectrum of tissue types, along with increased investigation into baseline functions of piRNA in human somatic tissue, will surely yield novel insights in this fast-growing field of non-coding RNA in cancer biology.
